# Data analysis and other considerations concerning the study of precipitation in Al–Mg–Si alloys by Atom Probe Tomography

**DOI:** 10.1016/j.dib.2015.09.045

**Published:** 2015-10-09

**Authors:** M.W. Zandbergen, Q. Xu, A. Cerezo, G.D.W. Smith

**Affiliations:** a Department of Materials, University of Oxford, Parks Road, Oxford OX1 3PH, UK; b National Center for HREM, Kavli Institute of Nanoscience, Delft University of Technology, Lorentzweg 1, NL-2628 CJ Delft, The Netherlands

## Abstract

Atom Probe Tomography (APT) analysis and hardness measurements were used to characterize the early stages of precipitation in an Al–0.51 at%Mg–0.94 at%Si alloy as reported in the accompanying Acta Materialia paper [1]. The changes in microstructure were investigated after single-stage or multi-stage heat treatments including natural ageing at 298 K (NA), pre-ageing at 353 K (PA), and automotive paint-bake ageing conditions at 453 K (PB). This article provides Supporting information and a detailed report on the experimental conditions and the data analysis methods used for this investigation. Careful design of experimental conditions and analysis methods was carried out to obtain consistent and reliable results. Detailed data on clustering for prolonged NA and PA treatments have been reported.

**Specifications Table**

**Table:** 

Subject area	*Materials Science*
More specific subject area	*Nanostructure, metallurgy, precipitation, aluminium alloys*
Type of data	*Table, image (APT, TEM), figure*
How data was acquired	*ATP, LEAP*
*ATP, LAR-3DAP*
*TEM, FEI Tecnai microscope*
Data format	*Analyzed*
Experimental factors	*Heat treatments at* 298 K, 353 K *and* 453 K
Experimental features	*Heat treatments were performed in an air furnace or oil bath. Samples for APT were made by electropolishing*
Data source location	*Department of Materials, Oxford University, Parks Road, Oxford, UK*
Data accessibility	*Data is in this article*

**Value of the data**

• The description of how the type of APT and the experimental settings influence detection of clusters and precipitation will be useful when comparing results obtained between different APT apparatus.
• Detailed description of which experimental parameters influence detection of clusters in ternary Al–Mg–Si by APT will help the community to design new experiments to measure precipitation in ternary Al–Mg–Si.
• The characterisation of the size and number density of precipitates and clusters during long ageing times may be used by the community to validate microstructure evolution models.
• The variation in composition of the precipitates and clusters can be used by the community to validate thermodynamic and kinetic databases for the Al–Mg–Si system.

## 1 Data, experimental design, materials and methods

### 1.1 Characteristics of the different stages of the precipitation in Al–Mg–Si alloys

See Table 1.

### 1.2 Experimental design

#### 1.2.1 Heat treatments and techniques

The composition of the investigated ternary Al–Mg–Si alloy is shown in Table 2. Sheets were cold-rolled to 1 mm thickness. All heat treatments were performed in an air furnace unless stated otherwise. The samples were solid solution heat-treated (SSHT) at 835 K for 30 min followed by a water quench to room temperature. Following SSHT, single or multi-stage ageing treatments were performed at 298 K (NA), 353 K (PA) and 453 K (PB). For multi-stage heat treatments including a PB treatment, the PB time was kept constant at 30 min. The precursor stages were systematically varied to mimic the different stages of industrial production. In some cases a transient heat treatment in an oil bath (referred to as a spike) was given for 10 s at 453 K before PA. The spike was given 1 min after the solid solution heat treatment (SSHT), and 2 min before PA. After the spike the material was water-quenched. Keeping the times constant between SSHT and the ageing treatments was important to get reproducible results. PB or PA treatments were commenced 1 or 3 min after the water quench, respectively. If necessary, the material was stored in liquid nitrogen after heat treatments to inhibit the influence of any subsequent NA. The various heat treatments are listed in Table 3.

Hardness measurements were carried out using a calibrated Vickers indenter at a 2 kg load and an indentation time of 10 s. 5 measurements were carried out across each sample to give a mean hardness value. The experimental error was estimated by dividing the standard deviation value by the square root of the number of measurements.

Needle-shaped specimens for APT experiments were made by a standard electropolishing method [33] from heat-treated thin bars by applying 10–15 V on the specimen in a 25% perchloric acid (65%) and 75% acetic acid (100%) solution at 278 K. These tips were then back-polished when necessary in a solution of 2% perchloric acid (65%) in 2-butoxyethanol. For APT analysis, two different types of 3D atom probe were used, a local-electrode atom probe (LEAP™) [34], [35] and a large-angle-reflectron 3D atom probe (LAR-3DAP™) [36]. Analyses were carried out at specimen temperatures of 25–30 K and pulse voltages of 15–20% of the standing DC voltage.

#### 1.2.2 APT analysis

Multiple samples were analysed for each heat treatment wherever possible. The size, morphology, and number density of the particles and the matrix compositions were extracted from the APT data by using particle selection software in PoSAP^™^ and IVAS^™^. Solute atoms (Mg and Si) were identified to be part of the same particle when they were within a maximum linear separation distance, *d*, from each other. A second parameter *N*_*min*_ was used, defined as the minimum number of solute atoms a particle had to contain for it to be identified as such. When a particle has fewer than *N*_*min*_ solute atoms, it was disregarded. Values of *d* and *N*_*min*_ were chosen so that no artefact solute “particles” were observed in a random solid solution of the same alloy composition. These values were determined by randomly assigning atom identities to the experimentally-observed positions in data sets and testing different *N*_*min*_ and *d* values on these randomized data. For LEAP measurements, a *d* of 0.65 nm and *N*_*min*_ of 10 solute atoms was used. As *N*_*min*_ was set at 10, particles smaller than 10 detected solute atoms were not selected. A *d* of 0.70 nm was allowed for LAR-3DAP measurements due to the lower detection efficiency of the LAR-3DAP (35% versus 50% for the LEAP). It should be noted that, due to these limited detection efficiencies, the particles contained 2–2.86 times as many solute atoms in reality as were detected in this work. The average size as detected by LAR-3DAP was adjusted by multiplying by 0.50/0.35 to correct for the difference in detection efficiency between LEAP and LAR-3DAP.

In some cases, further particle analysis was carried out using a *N*_*min*_ of 5. Lowering *N*_*min*_ and leaving *d* constant leads statistically to the selection of artefact “particles” in datasets with a random solute distribution. To take this into account, the relevant datasets were randomized to find the number density of artefact “particles” in the equivalent random solute distribution using a *N*_*min*_ of 5. An estimated number density for real particles consisting of more than 5 solute atoms was then calculated from the difference in number density between original and randomized datasets.

IVAS^™^ software was used to determine the dimensions of each particle measured along three orthogonal axes, *x*, *y*, and z. This analysis was only carried out on particles found using the parameters *d*=0.65 and *N*_*min*_=70 for LEAP measurements. The precipitate length corresponded to the longest (*z*) axis, e.g. the elongated direction of a β″ precipitate. For lath-shaped precipitates, the shortest (*x*) axis indicated the depth of the precipitates, whereas the (y) axis indicated the width of precipitates. The shortest (*x*) axis was used to indicate the diameter of particles that were needle-shaped. The statistical errors for number density of particles and particle Mg/Si ratios were calculated assuming a Poisson distribution for the particle population [33].

The measured overall solute concentrations by APT are shown in Table 2. The Mg concentration is underestimated by about 10%. The statistical error for the measured solute concentrations, based on the number of atoms in the dataset, was 0.01–0.02 at%. However, the systematic error was higher due to minor sample-to-sample composition variations and differences in experimental conditions and was estimated to be around 0.05 at%. The mean particle size was defined as the average number of solutes atoms in the particles. The error for this value was estimated by dividing the standard deviation of mean particles size by the square root of the number of particles. The statistical error for the average particle dimensions was similarly estimated by dividing standard deviation of average particle length by the square root of the number of particles.

Determination of the number density of particles according to their length was sometimes difficult, because some particles were cut off at the edges of the analysis. To take this into account, particles shorter than 6 nm or containing fewer than 100 solute atoms as measured by LEAP and cut off at the edges were discarded. To characterize the length of particles after 580 h PB (when the length of particles could not be determined by APT), transmission electron microscopy (TEM) was performed on a needle-shaped APT sample using a FEI Tecnai microscope at TU Delft.

#### 1.2.3 APT considerations

It should be noted that LEAP and LAR-3DAP measurements can give differences in results with respect to particle chemistry (by 20–30%) and number density (by 10–60%), especially in the cases of the very smallest particles [37]. These variations are due to differences in overall detection efficiency, field-of-view and mass resolution for the two types of atom probe. However, the trends in the data are the same in both atom probes. In general, the particle chemistries and number densities reported here are those detected by LEAP, to ensure full compatibility between data sets. The applied DC voltage also has a large influence on detection of these particles as the detected number density for the very smallest particles (containing fewer than 40 solute atoms) can decrease by as much as 20–30% with 1 kV of increase in voltage, due to limitations in spatial resolution [37]. Comparison of number densities of small particles was therefore only performed for measurements at similar low voltages (below 6 kV and within a 1 kV range).

Table 4 shows that the presence of large elongated particles (>15 nm) after prolonged PB led to a lower apparent overall solute concentration relative to the bulk alloy composition (decrease of 0.08–0.12 at% for Mg and 0.15–0.20 at% for Si). This is thought to occur due to failure in the detection of multiple ions evaporating per pulse from these larger precipitates [37], [38], [39], [40]. Relatively more Si atoms were undetected than Mg atoms. When the losses of Si and Mg were taken into account, the average Mg/Si ratios of large elongated particles (>15 nm) were estimated to be in the range of 0.90 and 1.05 as shown in Table 5. This is close to the Mg/Si ratio of Mg_5_Si_6_, but lower than the values of 1.2–1.4 found by APT without taking the losses of Mg and Si into account. In the other sections, only the unadjusted particle Mg/Si ratios as measured by APT are given. The average particle Mg/Si ratios of datasets containing large elongated particles (>15 nm) are therefore overestimated by 20–40% in this section. It should also be noted that local aberration effects due to ion trajectory differences between different phases [33] increase the apparently-measured width of the interface between matrix and precipitates by 2–3 nm. Only the apparent widths are given in the subsequent sections.

To estimate the relative Al content in particles, composition profiles of elongated particles were obtained by plotting a proximity histogram (proxigram) from isoconcentration surfaces defined so as to mark the matrix/particle interface [33]. An isosurface is a 3-D contour surface passing through all points of a particular solute concentration range. A 6–8 at% (Mg+Si) concentration value was used for large particles (average length >15 nm) and a 4–6 at% value for smaller particles (average length <15 nm). The delocalisation (smoothing) parameter was set at 3–5 nm.

### 1.3 Prolonged ageing

#### 1.3.1 Prolonged PB

**Fig. 1:**
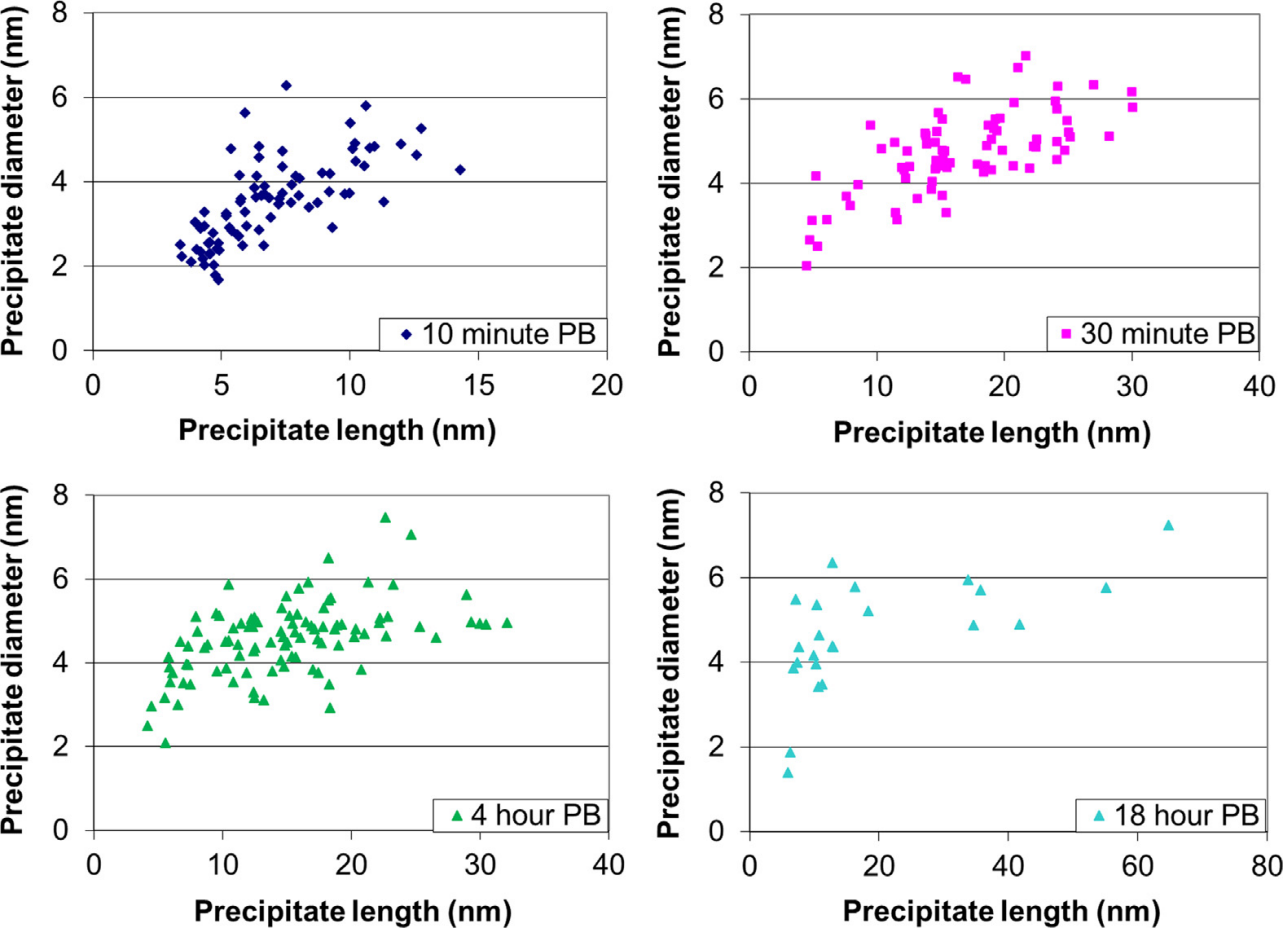
Measured diameters of precipitates plotted against precipitate length after various PB treatments at 453 K.

The data shown here are complimentary to the results discussed in the accompanying Acta Materialia paper [1]. Distributions of the diameter and the detected precipitate Mg/Si ratio against the precipitate length are plotted in Fig. 1. From the APT analysis observations, there is no clear distinction between the successive phases, the change in precipitate length being continuous. The diameter increases from 3–4 nm for short-elongated precipitates to 5–6.5 nm for precipitates longer than 15 nm. The diameter of the precipitates increases only slightly (by <1 nm) as they grow longer than 15 nm, demonstrating that the large needle-shaped precipitates hardly coarsen laterally with increasing ageing time.

**Fig. 2:**
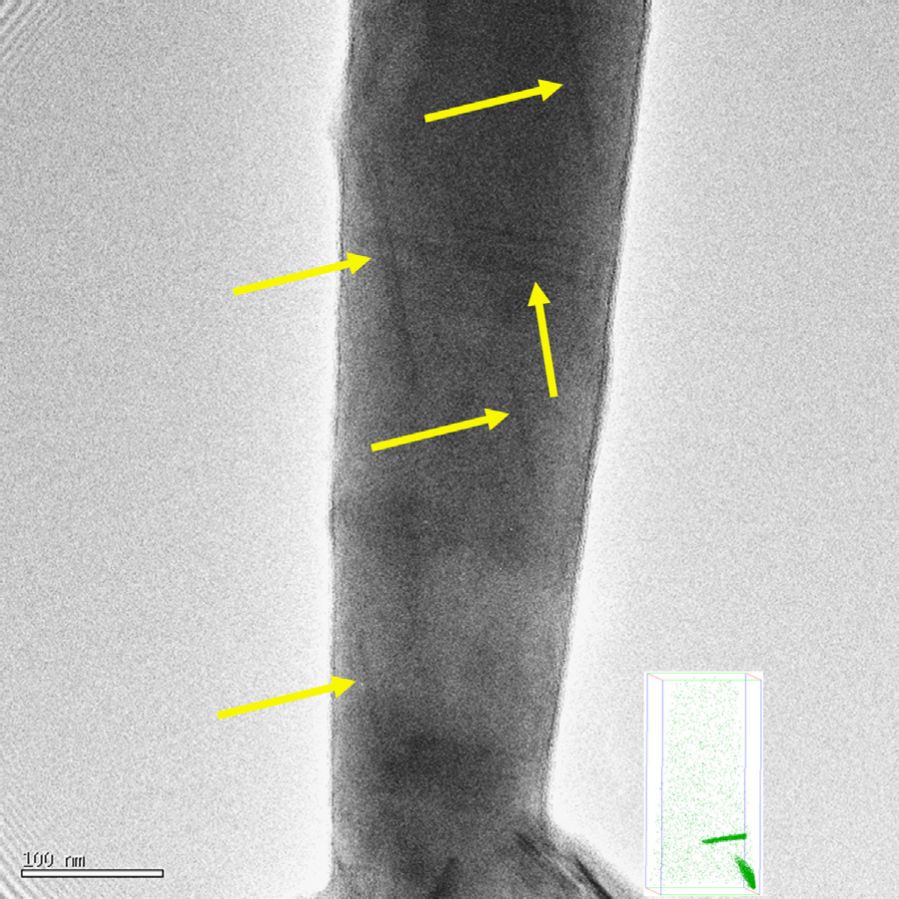
TEM image of APT specimen heat treated for 1 min at room temperature and 580 h at 453 K. The yellow arrows indicate elongated B′ precipitates. The inset is a LAR-3DAP measurement of the same material at the same magnification, but not the same area of analysis.

Ageing for 580 h at 453 K gives precipitates of 100–200 nm in length as shown in the TEM image in Fig. 2. APT measurements show that these precipitates are lath-shaped. The precipitates have probably transformed into B′, which has a proposed composition of Mg_9_Al_3_Si_7_ [30], [41]. The proposed B′ Mg/Si ratio of 1.29 is close to that found here after adjusting for the preferential loss of solutes (1.55–1.75 as measured by APT to 1.0–1.4 after adjustment for loss of Mg and Si atoms).

**Fig. 3:**
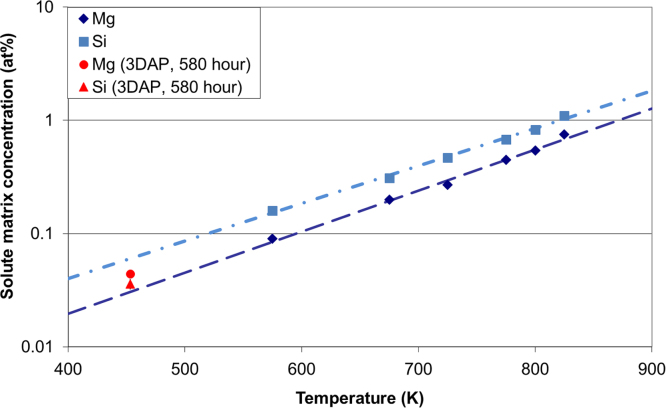
Solid solubility limits (in at%) of Mg and Si in aluminium in Al–Mg–Si system. Values on the dark blue and pink lines are from [42].

After prolonged PB, the solute matrix concentrations decrease to below 0.05 at%. Fig. 3 shows that extrapolations of solid solubility limits at temperatures above 498 K known from the literature [42] coincide with the measured matrix concentrations after 580 h PB. This demonstrates that the solid solubility limits of Mg and Si are below 0.05 at% at 453 K in ternary Al–Mg–Si, meaning that 0.48 at% Mg and 0.89 at% Si have come out of solution. The B′ precipitates are Mg-rich, showing that 0.4–0.6 at% Si is not accounted for. Consequently, it is possible that large Si-rich precipitates have formed, as has been reported to occur in excess-Si Al–Mg–Si alloys after prolonged ageing at 453 K (>100 h) [43], [44]. The Si diffusion distances at equilibrium vacancy concentrations are estimated to be above 700 nm for 400 h ageing at 453 K [45]. Although large Si precipitates have not been observed in the APT measurements, it is possible that they were formed during prolonged PB as the longest experimental run was only 60 nm wide and 350 nm in length.

#### 1.3.2 Prolonged NA

**Fig. 4:**
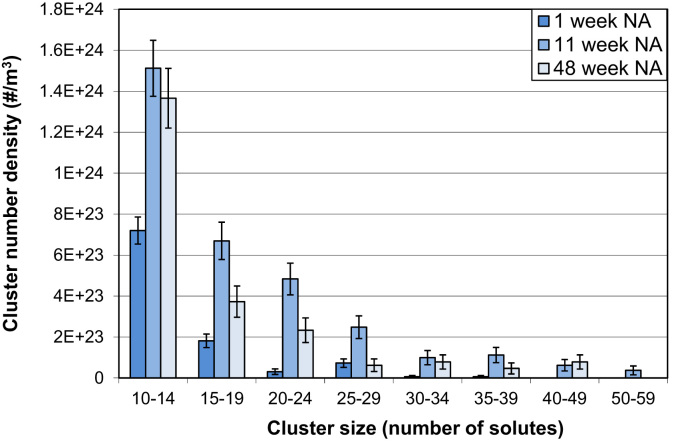
Cluster number densities for different cluster size ranges after 1, 11 or 48 weeks NA as measured by LAR-3DAP. The cluster sizes were obtained using a *N*_*min*_ of 10 for cluster analysis.

The data shown here are complimentary to the results discussed in the accompanying Acta Materialia paper [1]. Detailed APT data after prolonged NA by LAR-3DAP are shown in Table 6 and Fig. 4. After 11 or 48 weeks of NA, the cluster number density is stable. The clusters grow in size from 1 week to 11 weeks NA. The average cluster Mg/Si ratio and cluster Mg/Si ratio distributions do not change.

#### 1.3.3 Prolonged PA

**Fig. 5:**
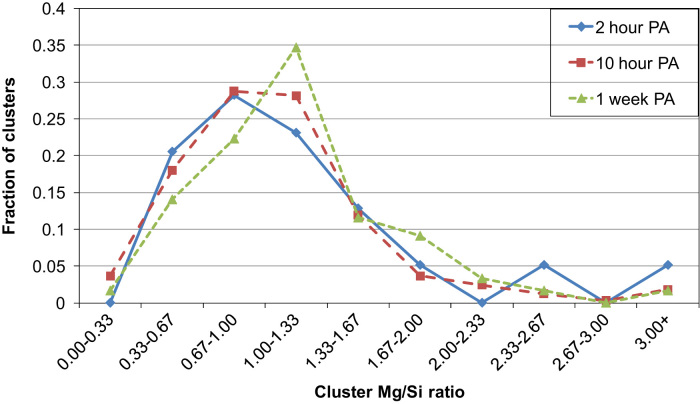
Fraction of clusters plotted against cluster Mg/Si ratio ranges after 2 or 10 h or 1 week PA as measured by LAR-3DAP.

**Fig. 6:**
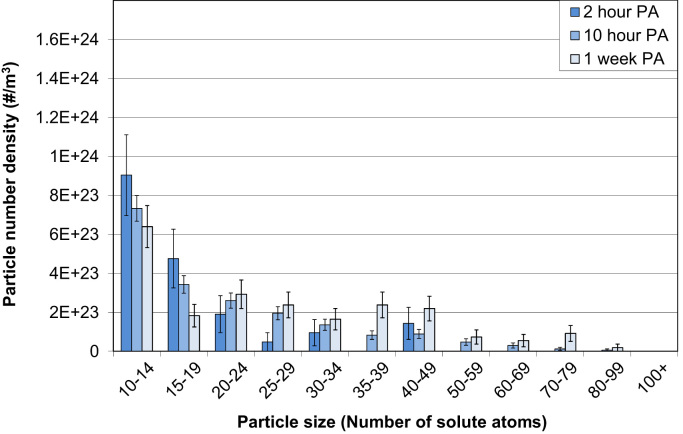
Particle number densities for different particle size ranges after 2 or 10 h or 1 week PA as measured by LAR-3DAP. A *N*_*min*_ of 10 was used for particle analysis.

APT data after prolonged PA by LAR-3DAP are shown in Table 7 and Fig. 5, Fig. 6. Clusters continue to increase in size from 2 h to 1 week PA. After 2 h PA, the cluster number density as measured by LAR-3DAP hardly increases with PA time (<25%), but the average cluster size does by 60% from 2 h to 1 week PA.

#### 1.3.4 Spike and prolonged pre-ageing

**Fig. 7:**
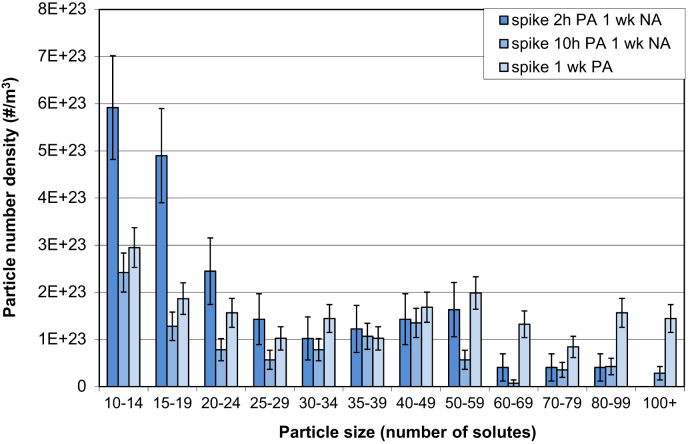
Particle number densities for different particle size ranges after a spike and 2 or 10 h or 1 week PA as measured by LAR-3DAP. A *N*_*min*_ of 10 was used for particle analysis.

**Fig. 8:**
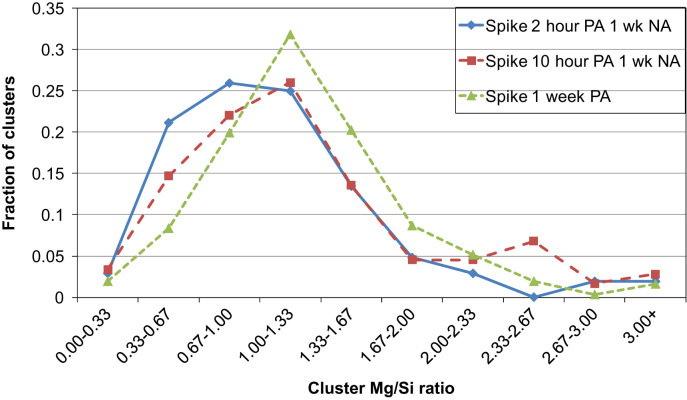
Fraction of clusters plotted against cluster Mg/Si ratio ranges after a spike followed by 2 or 10 h or 1 week PA as measured by LAR-3DAP.

**Fig. 9:**
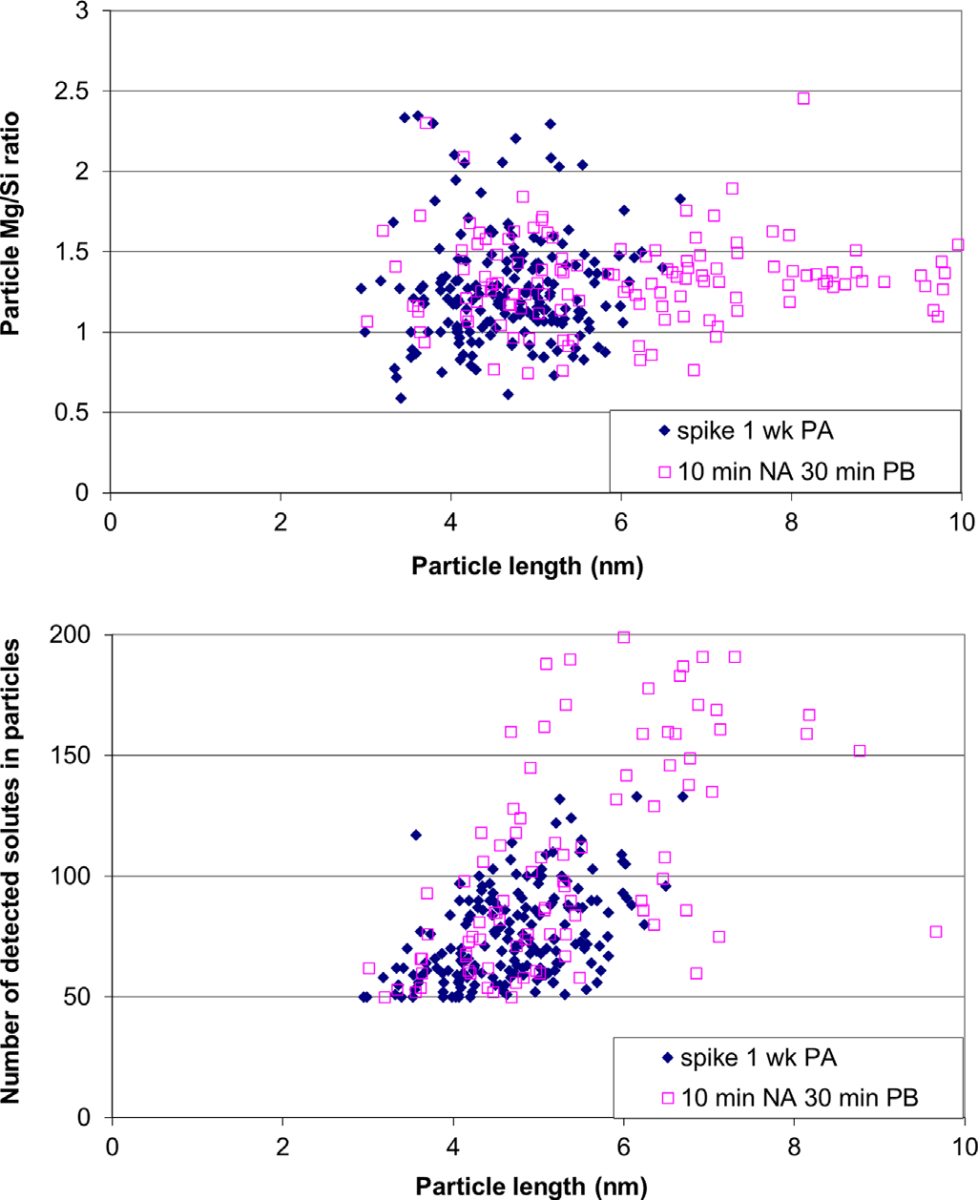
Particle Mg/Si ratio or number of detected solute atoms in particles plotted against particle length after a spike and 1 week PA (blue) or 10 min NA and 30 min PB (pink) as measured by LAR-3DAP. Only particles consisting of 50 solute atoms or more are taken into account. The particle sizes and compositions were obtained using a *N*_*min*_ of 10 for particle analysis.

**Fig. 10:**
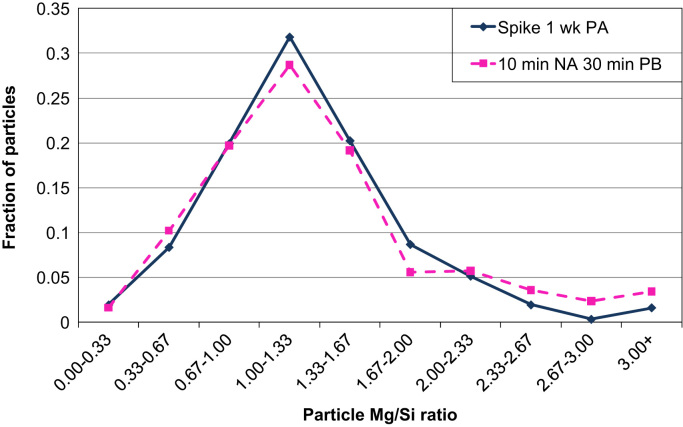
Fraction of particles containing fewer than 150 solute atoms plotted against particle Mg/Si ratio ranges after a spike followed by 1 week PA or after 10 min NA and a 30 min PB as measured by LAR-3DAP.

APT measurements show that application of a spike (10 s at 453 K) before PA leads to larger clusters with similar Mg/Si ratios to those formed without a spike. Similar to the results by LEAP as discussed in the accompanying Acta Materialia paper [1], LAR-3DAP measurements show that application of a spike before PA leads to larger clusters with similar Mg/Si ratios to those formed without a spike, as illustrated in Table 7 and Fig. 7, Fig. 8. After 1 week PA, clusters are identical in size, morphology and chemistry to the spheroidal precipitates present after PB, as shown in Fig. 9, Fig. 10. Therefore, applying a spike has two effects: it increases the number density of larger clusters which are close in size and chemistry to spheroidal precipitates and it decreases the number density of smaller ones.

#### 1.3.5 Clusters after PB

The effects of ageing at 453 K after NA and PA on clusters and precipitation is discussed in the accompanying Acta Materialia paper [1]. Table 8 summarises the change in number densities of clusters and precipitates after different heat treatments. Not every cluster formed during PA grows into an elongated precipitate upon subsequent PB. For 10 h PA, the decrease in number density of small clusters is ~90×10^22^/m^3^ during PB, whereas the increase in precipitates containing more than 70 solute atoms is only 20–30×10^22^/m^3^. Clusters present after a 10 or 30 min PB have a Mg/Si ratio of 0.9–1.2, which is comparable to that of clusters present after PA before the PB and to that of precipitates formed during the PB.

### 1.4 Additional data after multiple heat treatments

#### 1.4.1 Effect of pre-ageing on the paint-bake

The data shown here is complimentary to the results discussed in the accompanying Acta Materialia paper [1]. When PA is given prior to NA, the change in hardness during 1 week NA is significantly reduced. The hardness is 55–60 *H_v_* after 2 h PA and increases to 60–67 *H_v_* after 1 week NA. 10 h PA leads to a hardness of 67±1 *H_v_*, which hardly increases during 1 week of NA.

**Fig. 11:**
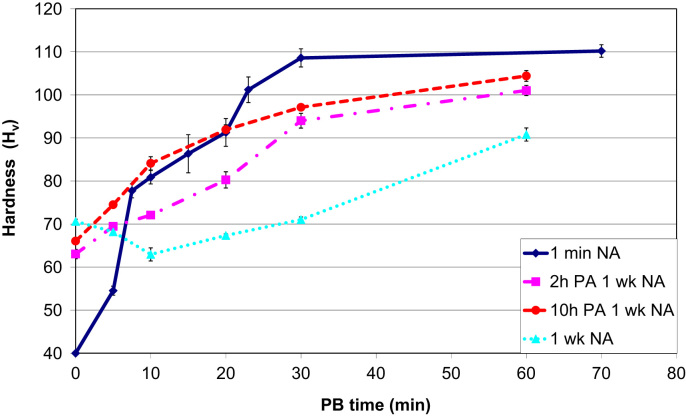
Change in hardness with PB time after 1 min NA or 1 week NA when no PA is given or when 2 or 10 h of PA is given prior to NA.

PA partially mitigates the deleterious effects of subsequent 1 week NA as illustrated in the hardness plots in Fig. 11. The hardness increases by approximately 30 *H_v_* after 30 min PB when PA is given, which is lower than the 64±3 *H_v_* increase when the PB is given 1 min after the SSHT, but better than the PB response after NA without PA. 1 week NA after 2 h PA has little effect on the PB response, i.e. hardly any difference is seen in the hardness curves after 2 h PA with and without 1 week NA. Increasing the PA time to 10 h improves the hardening response by 5–10 *H_v_* during the first 20 min of PB.

**Fig. 12:**
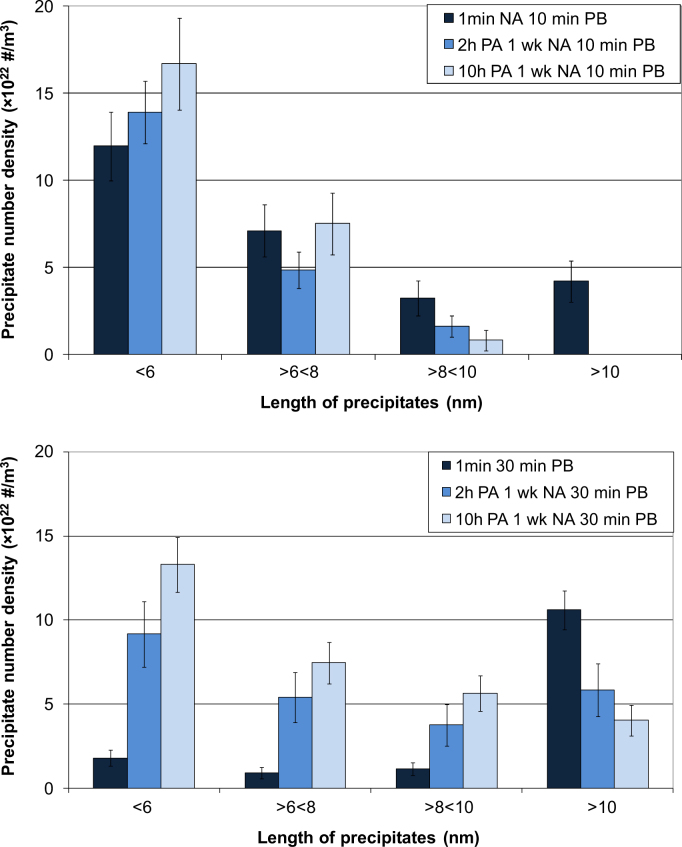
Number density of precipitates for different precipitate lengths after 1 min NA and a 10 or 30 min PB; 2 h PA 1 week NA and a 10 or 30 min PB; or 10 h PA 1 week NA and a 10 or 30 min PB.

When PA is given, the PB response is slower than when the PB is given directly after the SSHT. After 10 min PB, the microstructure is comparable due to the fact that clusters, precursors to the elongated precipitates, are already present when PA is given. However, precipitates grow considerably faster between 10 and 30 min PB when the NA time is restricted to 1 min as shown in Fig. 12.

### 1.5 Calculation of the nucleation barrier after NA

After 100 min NA, the percentage of total Mg atoms of the alloy in clusters is limited to 5–6%. It should be noted that the percentage of Mg in clusters might be underestimated as some clusters are too small to be detected by APT. If we make a conservative estimate that we only measure a third of the smallest clusters then 15–18% of Mg should be in clusters, corresponding to a decrease in the Mg concentration of ~0.08 at%. This decrease in matrix Mg concentration increases the nucleation barrier (ΔG^*^). ΔG^*^ for spherical particles, according to the classical nucleation and growth theory [46], is estimated by: ΔG*=16*πγ*^3^/3Δ*g*^2^, where Δ*g* is the driving force per unit volume for precipitation, *γ* the specific interfacial energy between the matrix and cluster or precipitate (between 0.1 and 0.45 J/m^2^ for β″ [47]). Δ*g* can be estimated (assuming the strain energy per unit volume is negligible) by: Δ*g*=Δ*g*_*chem*_=−(*k_B_T*/*V*_*β*_)ln(*C*/*C*_*eq*_), where *k_B_* is the Boltzmann constant (J/K), *T* the temperature (K), *V_β_* the atomic volume of phase β (m^3^) and *C_eq_* the equilibrium matrix solute concentration in the solvent. A *V_β_* for the β″ phase is used for all calculations. Table 9 shows the equilibrium matrix solute concentrations for different phases determined by APT in this work. These concentrations have been used to calculate Δ*g* and ΔG^*^ for clusters with a chemistry close to that of elongated precipitates (PB clusters) as shown in Table 10. Two values for *γ*, 0.15 or 0.3 J/m^2^, have been investigated. The solute concentration decrease in the matrix after 100 min NA leads to an increase of ~20% for ΔG^*^ for clusters acting as precursors to the elongated phases. The relative change in nucleation rate is calculated by dividing exp(−ΔG^*^/*k_B_T*) after 100 min NA by that before NA. For both *γ* values, ΔG^*^ increases after 100 min NA leading to a decrease in the nucleation rate. It should be noted that the values for ΔG^*^ are reasonable at 0.70 or 0.85 eV when *γ* is 0.15 J/m^2^. This value is comparable to 0.75–0.90 eV (72–87 kJ/mol) as reported by Banhart et al. [48] based positron annihilation lifetime spectroscopy (PALS) measurements. The calculations show that the relatively small changes in matrix solute concentrations after 100 min NA lead to a significant reduction of nucleation of clusters, which develop into larger precipitates.

## Figures and Tables

**Table 1:** Characteristics of phases observed in Al–Mg–Si alloys as reported in the literature.

**Type**	**Composition**^a^	**Unit cell**	**Morphology**	**Ref.**
Clusters	Mg/Si: 1–2	–	Spherical	[2], [3], [4], [5], [6], [7], [8], [9], [10], [11], [12], [13], [14], [15], [16]
GP zones/ Initial β″/ Pre- β″	Mg*_x_*Al_5−*x*_Si_6_ or Mg_2+x_Al_7-x-y_Si_2+y_	Monoclinic, C2/m, *a*=1.48, *b*=0.405, *c*=0.648 nm; *β*=105.3°	Spherical 1–3 nm/needles of 2×2×20 nm	[17], [18], [19], [20], [21], [22], [23]
β″	Mg_5_Si_6_	Monoclinic, C2/m, *a*=1.516, *b*=0.405, *c*=0.674 nm; *β*=105.3°	Needles of ~4×4×50 nm	[24], [25], [26]
β′	Mg_1.8_Si	Hexagonal, P6_3_, *a*=0.715, *c*=0.405/1.215 nm; *γ*=120°	Needles >100 nm long, 10 nm in diameter	[18], [20], [27]
U1 (Type A)	MgAl_2_Si_2_	Trigonal, P_-3m1_, *a*=0.405, *c*=0.674 nm; *γ*=120°	Needles >100 nm long, 15 nm in diameter	[20], [28], [29]
U2 (Type B)	MgAlSi	Orthorhombic, P_nma_, *a*=0.675, *b*=0.405, *c*=0.794 nm	Needles >100 nm long, 15 nm in diameter	[20], [29]
B′ (Type C)	Mg_9_Al_3_Si_7_	Hexagonal, *a*=1.03 nm, *c*=0.405 nm	Lath-shaped	[30]
β	Mg_2_Si	FCC, CaF, *a*=0.639 nm	Plates or Cubes of 10–20 μm	[31], [32]

a The Al content of the early-stage phases is very difficult to determine and is therefore not given here.

**Table 2:** Composition of the alloy in wt% and at%.

	**Al**	**Si**	**Mg**	**Cu**	**Fe**	**Mn**
(wt%)	Balance	0.98±0.02	0.46±0.01	0.029±0.002	0.17±0.01	0.10±0.02
(at%)	Balance	0.94±0.02	0.51±0.01	0.013±0.001	0.08±0.01	0.05±0.01
Measured by APT						
(at%)	Balance	0.95±0.01	0.46±0.01	0.016±0.001	–	0.03±0.01

**Table 3:** Heat treatments.

Heat treatment	
1 min NA+PB	1 min natural ageing at 298 K+10 min, 30 min, 4 h, 18 h, 400 h, or 580 h ageing at 453 K
NA	100, 1000 and 10,000 min natural ageing at 298 K
PA (+ NA)	2 h, 10 h or 1 week pre-ageing at 353 K (+ 1 week natural ageing at 298 K)
Spike+PA+NA	10 s at 453 K+2 h or 10 h pre-ageing at 353 K+1 week natural ageing at 298 K
NA+PB	1 min, 10 min, 100 min or 1 week natural ageing at 298 K+30 min ageing at 453 K
2 or 10 h PA+1 week NA+PB	2 h or 10 h pre-ageing at 353 K+1 week natural ageing at 298 K+30 min ageing at 453 K
Spike+2 h PA+NA+PB	10 s at 453 K+2 h or 10 h pre-ageing at 353 K+1 week natural ageing at 298 K+30 min ageing at 453 K

**Table 4:** Lower detected overall solute concentration by LEAP after 30 min or 18 h ageing at 453 K.

	**Mg**	**Si**
Average overall Mg and Si concentrations of measurements after NA or PA (at%)^a^	0.46±0.01	0.95±0.01
Overall Mg and Si concentrations of measurement after 30 min PB^b^ (at%)	0.36±0.01	0.77±0.01
**Loss of Mg and Si atoms in 30 min PB measurement (at%)**	**0.10**	**0.18**
Overall Mg and Si concentrations of measurement after 18 h PB^b^ (at%)	0.34±0.01	0.79±0.01
**Loss of Mg and Si atoms in 18 h PB measurement (at%)**	**0.12**	**0.16**

a The average overall composition of 10 LEAP measurements after NA or PA. These datasets only contain clusters (particles containing fewer than 70 solute atoms).

b Mainly elongated precipitates longer than 15 nm are present.

**Table 5:** Estimation of precipitate Mg/Si ratio compensating for the loss of Si and Mg atoms during APT analysis.

	**Mg**	**Si**
Average overall Mg and Si concentrations of measurements after NA or PA (at%)^a^	0.46±0.01	0.95±0.01
Matrix Mg and Si concentrations of measurement after 30 min PB^b^ (at%)	0.16±0.01	0.62±0.01
**Estimated amount of solutes in elongated precipitates in 30 min PB measurement (at%)**	**0.30**	**0.33**
**Estimated average precipitate Mg/Si ratio**	**0.91**	
**Average precipitate Mg/Si ratio as measured by LEAP**	**1.35**	
Matrix Mg and Si concentrations of measurement after 18 h PB^b^ (at%)	0.09±0.01	0.60±0.01
**Estimated amount of solutes in elongated precipitates in 18 h PB measurement (at%)**	**0.37**	**0.35**
**Estimated average precipitate Mg/Si ratio**	**1.06**	
**Average precipitate Mg/Si ratio as measured by LEAP**	**1.35**	

a The average overall composition of 10 LEAP measurements after NA or PA. These datasets only contain clusters (particles containing fewer than 70 solute atoms).

b Mainly elongated precipitates longer than 12 nm are present.

**Table 6:** LAR-3DAP measurements after 1, 11 or 48 week NA.

NA time	Average cluster size (number of solute atoms)	Cluster Mg/Si ratio^a^	Cluster number density (×10^22^/m^3^) *N*_*min*_=10	Cluster number density (×10^22^/m^3^) *N*_*min*_=5
**1 week NA**	13.9±0.5	0.86±0.03	120±13	400±50
**11 week NA**	17.8±0.9	0.87±0.02	341±21	490±50
**48 week NA**	16.4±0.8	0.95±0.03	223±19	400±50

a The variations in cluster chemistry are assumed to be mainly caused by the differences in measured overall composition.

**Table 7:** LAR-3DAP measurements after 2 or 10 h or 1 week PA with and without a spike.

Heat treatment	Average particle size (number of solute atoms)	Particle Mg/Si ratio^a^	Cluster^b^ number density (×10^22^/m^3^)	Precipitate^b^ number density (×10^22^/m^3^)
**Without spike**				
2 h *PA*	18.2±2.4	1.02±0.05	186±30	–
10 h *PA*	22.0±0.7	0.93±0.02	184±11	9±2
1 *week PA*	28.6±2.1	1.06±0.03	197±18	24±6
**With spike**				
*Spike* 2 h *PA* 1 *week NA*	26.7±1.6	0.98±0.03	184±18	29±8
*Spike* 10 h *PA* 1 *week NA*	34.2±2.0	1.11±0.02	108±10	17±4
*Spike* 1 *week PA*	45.6±1.8	1.19±0.01	116±10	72±7

a The variations in cluster chemistry are assumed to be mainly caused by the differences in measured overall composition.

b Precipitates are particles containing more than 50 solute atoms as measured by LAR-3DAP, clusters contain fewer than 50 solute atoms (*N_min_* was 10 for particle analysis).

**Table 8:** Number densities of particles containing 10–20, 20–40, 40–70 and 70+ solute atoms as measured by LEAP after different heat treatments using a *N*_*min*_ of 10.

Heat treatment	Number density clusters of 10–20 solute atoms (×10^22^/m^3^)	Number density clusters of 20–40 solute atoms (×10^22^/m^3^)	Number density clusters of 40–70 solute atoms (×10^22^/m^3^)	Number density precipitates 70+ solute atoms (×10^22^/m^3^)
**1 min NA**				
**10 min PB**^a^	34±3	22±3	10±2	26±2
**30 min PB**	4.7±0.7	1.2±0.4	0.7±0.3	13±1
**1 week NA**				
**Before PB**	101±6	15±2	–	–
**30 min PB**	85±5	30±3	2±1	1±1
**10 h PA 1 week NA**				
**Before PB**	85±12	73±11	13±5	1±1
**10 min PB**	45±4	26±3	17±3	24±3
**30 min PB**	45±3	21±2	9±1	30±2
**Spike 2 h PA 1 week NA**				
**Before PB**	59±5	26±3	12±2	3±1
**30 min PB**	24±2	12±2	9±1	27±3

a This was measured at 1–2 kV lower voltage than the other measurement.

**Table 9:** Matrix solute concentrations below which certain phases are not observed at 453 K.

Phase	Matrix Mg concentrations (at%)	Matrix Si concentration (at%)
NA clusters^a^	0.49±0.02	0.92±0.03
PA/PB clusters	0.080±0.002	0.58±0.02
β″	0.043±0.003	0.046±0.006

a Clusters dissolve or change chemistry and size upon ageing at 453 K.

**Table 10:** Estimation of nucleation barriers and the decrease in nucleation rate after 100 min NA.

	*γ* (J/m^2^)		Δ*g*_*chem*._ (J/m^3^)	ΔG^*^ (J)	Exp(ΔG^*^/kT)	Exp(ΔG^*^/kT)_100__ min*NA*_/Exp(ΔG^*^/kT)*_noNA_*
**Clusters formed during PB**	0.3	Before NA	−7.1×10^8^	9.0×10^−19^	3.4×10^−63^	1.4×10^−13^
	100 min NA	−6.5×10^8^	1.1×10^−18^	4.9×10^−76^	
0.15	Before NA	−7.1×10^8^	1.1×10^−19^	1.6×10^−8^	2.5×10^−2^
	100 min NA	−6.5×10^8^	1.4×10^−19^	3.9×10^−10^	
